# Supplementary material for: Mechanism of SOS PR-domain autoinhibition revealed by single-molecule assays on native protein from lysate
Source: Nat Commun. 2017 Apr 28;8:15061. doi: 10.1038/ncomms15061 (PMC5414354; doi:10.1038/ncomms15061)
Supplement: Supplementary Information — Supplementary Figures, Supplementary Table and Supplementary Reference [file ncomms15061-s1.pdf]

Supplementary Information

Mechanism of SOS PR-domain autoinhibition revealed by single-molecule assays on native protein from lysate

Young Kwang Lee[1,2], Shalini T. Low-Nam[1], Jean K. Chung[1], Scott D. Hansen[1], Hiu Yue Monatrice Lam[1], Steven Alvarez[1], Jay T. Groves*[1,2]

[1]Department of Chemistry, University of California, Berkeley, California 94720, USA
[2]Molecular Biophysics & Integrated Bioimaging Division, Lawrence Berkeley National Laboratory, Berkeley California 94720, USA

Correspondence should be addressed to J.T.G. (jtgroves@lbl.gov).

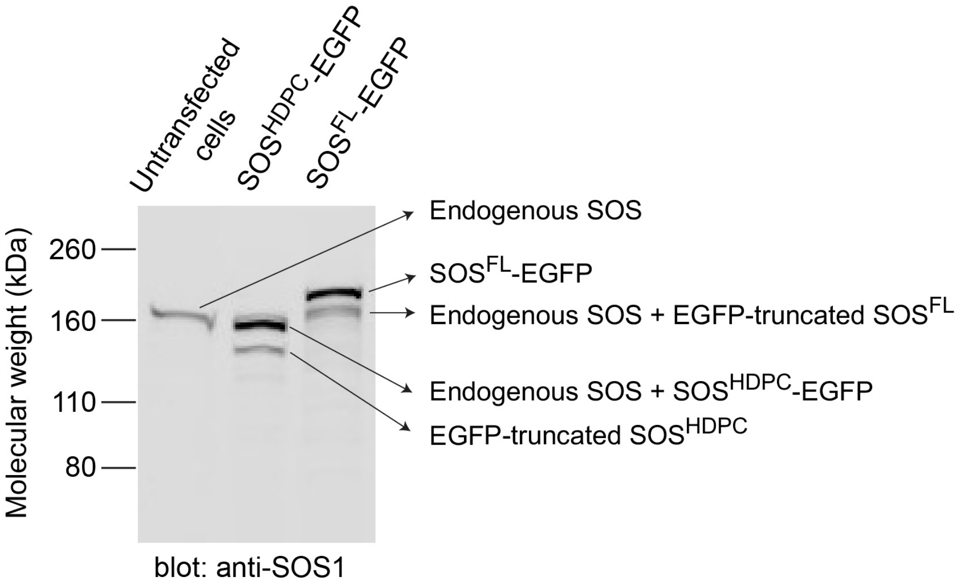

Supplementary Figure 1. Western blots of endogenous and ectopically expressed SOS. Ectopically-expressed SOS was overexpressed compared to endogenous SOS in HEK293T cells. Anti-SOS1 antibody specific for an N-terminal epitope was used for western blot. An expression level of endogenous and ectopic SOS was compared based on corresponding band intensities in western blots. We observed that a similar degree of ~20 kDa C-terminal truncation occurred for SOS constructs fused with C-terminal EGFP. This truncation is restricted within a region of EGFP (27 kDa). Hence, the C-terminal truncated constructs have the intact SOS domains (henceforth referred to as EGFP-truncated SOS constructs). An endogenous SOS band overlapped with other bands, i.e. SOS^{HDPC}-EGFP and EGFP-truncated SOS^{FL}. This makes it difficult to determine the band intensity of endogenous SOS and compare it with that of ectopic SOS in the same lane. Hence we determined the endogenous SOS intensity in a separate lane loaded with untransfected cell lysate. The net band intensity of ectopic SOS was determined by subtracting the endogenous SOS intensity from the whole SOS intensity in each lane. We estimate the expression of SOS^{HDPC} and SOS^{FL} to be approximately 5-10-fold higher than

endogenous SOS. SOS^{Cat} and SOS^{CatPR} could not be detected using the anti-SOS antibody due to lack of the N-terminal epitope. Using EGFP fluorescence measurements, SOS^{Cat} and SOS^{CatPR} were estimated to be 3-5-fold more highly expressed than SOS^{FL} and SOS^{HDPC} (see also, Supplementary Fig. 2). The amount of total proteins loaded in each lane was measured by Bradford assay. Untransfected cell lysate was loaded in 3-fold excess with respect to transfected cell lysate to clearly visualize endogenous SOS that exists at a low level, and the endogenous SOS band intensity was normalized to the amount of total proteins loaded for transfected cell lysate.

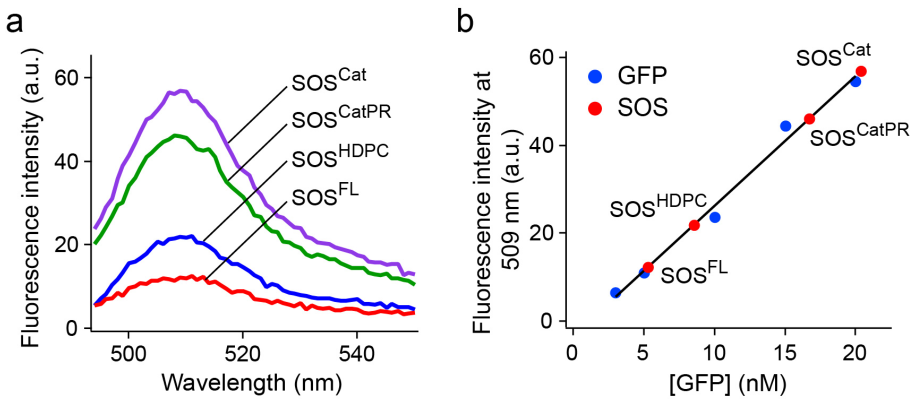

Supplementary Figure 2. Quantification of SOS-EGFP in cell lysate. Concentrations of SOS-EGFP in transfected cell lysate were determined using a fluorescence standard curve based on EGFP diluted in untransfected cell lysate. First, concentration of total proteins in each transfected cell lysate was determined by Bradford assay. Each lysate was diluted with lysis buffer to ensure equal concentrations of total protein. Fluorescence spectra of SOS-EGFP in cell lysate were measured, as shown in **a**. Emission intensity at 509 nm was monitored to estimate concentrations of SOS-EGFP, as shown in **b**.

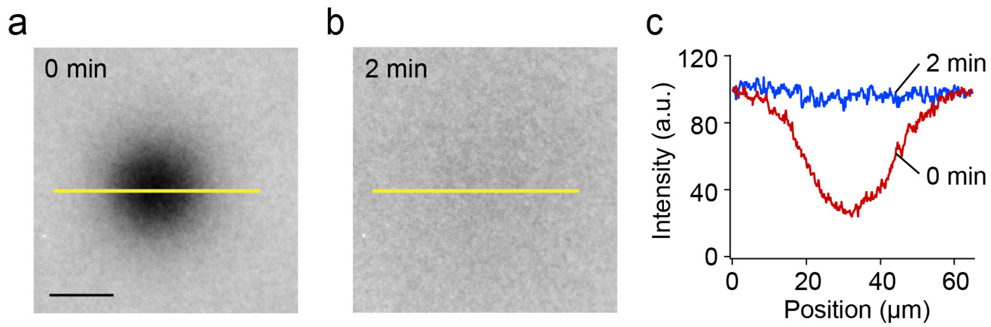

Supplementary Figure 3. Fluorescence recovery after photobleaching (FRAP) experiments of membrane-tethered Ras. (**a**) Ras loaded with Atto 488-GppNp was photobleached by intense illumination of a 488 nm laser. (**b**) After 2 min the bleached area was completely recovered and showed a uniform fluorescence. (**c**) Intensity profiles (yellow lines) for images in **a** and **b**. Scale bar: 20 μm.

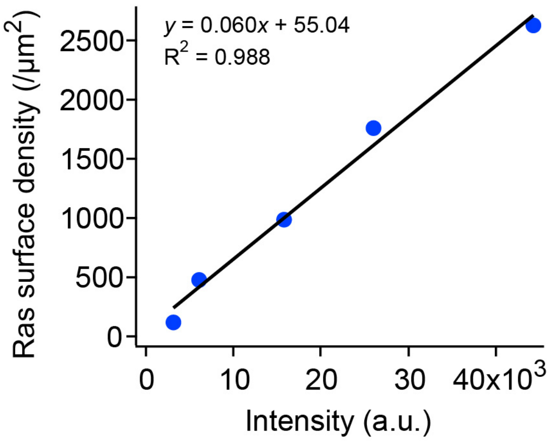

Supplementary Figure 4. Calibration curve for quantification of Ras density by epifluorescence imaging. Ras density was determined by fluorescence correlation spectroscopy (FCS) using samples loaded with a fluorescent nucleotide analog (Atto 488-GppNp) and correlated with epifluorescence images.

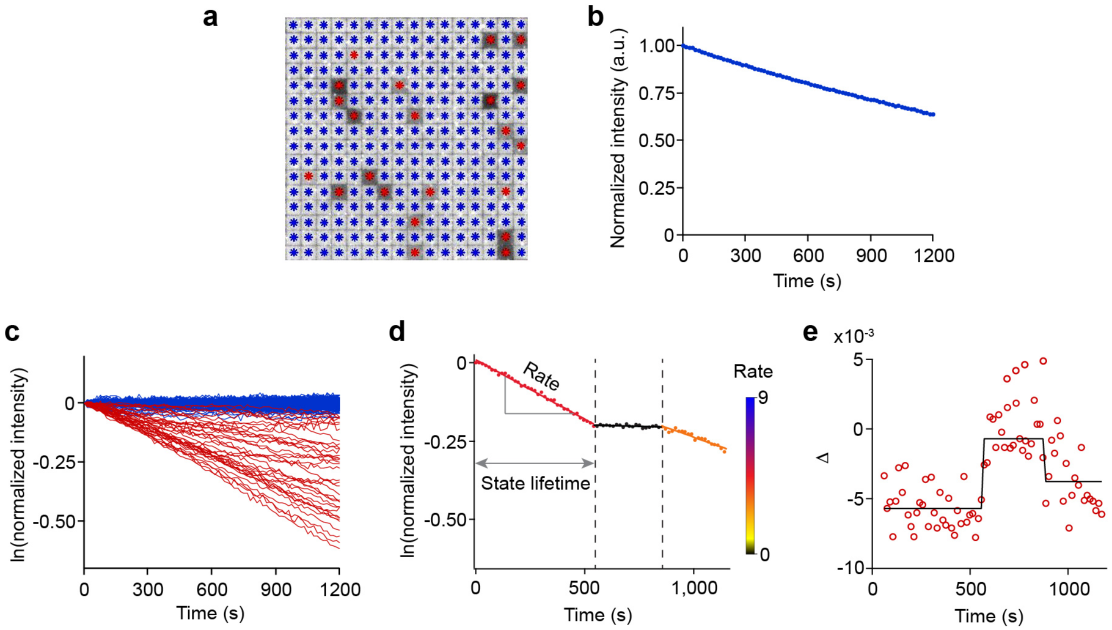

Supplementary Figure 5. Analysis approaches for single-molecule SOS activity assays. (**a**) Active (with SOS; red asterisks) and inactive corrals (without SOS; blue asterisks) were discriminated based on intensity at the assay endpoint. (**b**) Intrinsic Ras turnover and photobleaching factors were calculated from the average fluorescence intensity trace of inactive corrals. Fluorescence decay rate of active corrals is a convolution of catalytic SOS turnover (k_{sos}), intrinsic Ras turnover (k_i) and photobleaching (k_p). All traces were divided by the average intensity trace of inactive corrals to remove contributions from intrinsic nucleotide exchange and photobleaching. Mathematical description of this process to calculate fluorescence decay per SOS, $I_{SOS}(t)$, is as follows:

$I_{SOS}(t) = \frac{I_0 e^{-(k_{sos}+k_p+k_i)\cdot t}}{I_0 e^{-(k_p+k_i)\cdot t}} = e^{-k_{sos}\cdot t}$. k_{sos}, k_p and k_i represent SOS turnover, photobleaching and intrinsic Ras turnover, respectively. (**c**) The catalytic exchange kinetics of all corrals. Blue and red traces represent inactive and active corrals, respectively. (**d**) A representative single enzyme activity trace. (**e**) Distinct functional activity states were detected using the change point detection algorithm[1]. The trace in **d** was recast in the form of the local slope of the kinetic traces (red circles) calculated as the difference between consecutive data points ($\Delta=(x_i+1-x_i)/(t_i+1-t_i)$), where i is a running variable looping through the trace and x_i denotes the data point i. The result of the change point identification is shown as black solid lines).

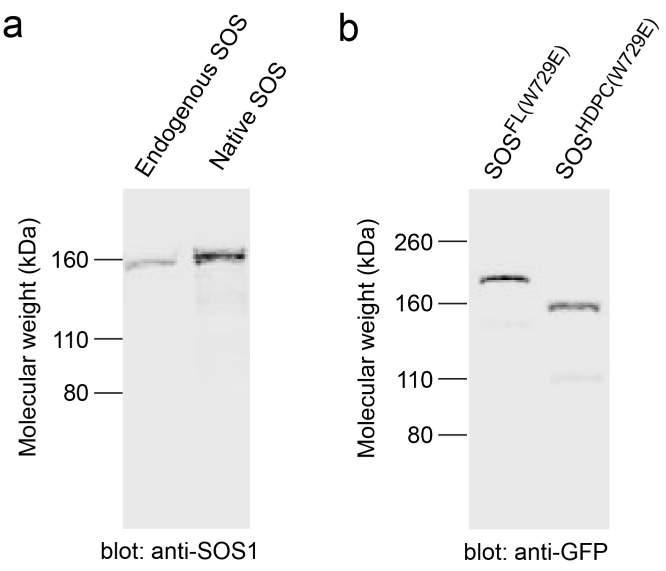

Supplementary Figure 6. Western blots of native SOS and W729E SOS mutants. (a) Anti-SOS1 western blot of endogenous SOS and ectopically-expressed native SOS. (b) Anti-GFP western blot of $SOS^{FL(W729E)}$ and $SOS^{HDPC(W729E)}$. A W729E point mutation disrupts the allosteric site of SOS.

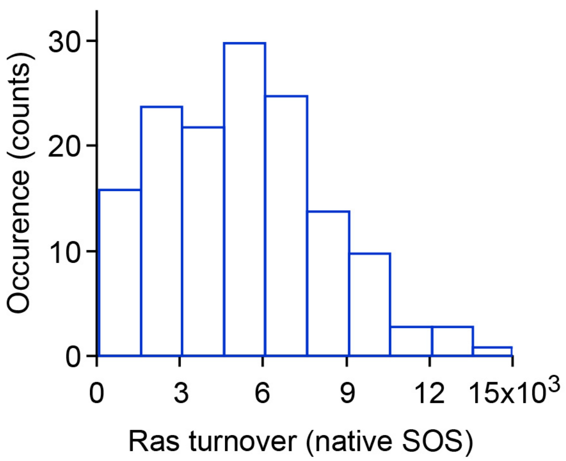

Supplementary Figure 7. Histogram of total Ras turnovers by individual native SOS enzymes. Total Ras turnovers were measured from the single-molecule SOS activity assay shown in **Figure 2**.

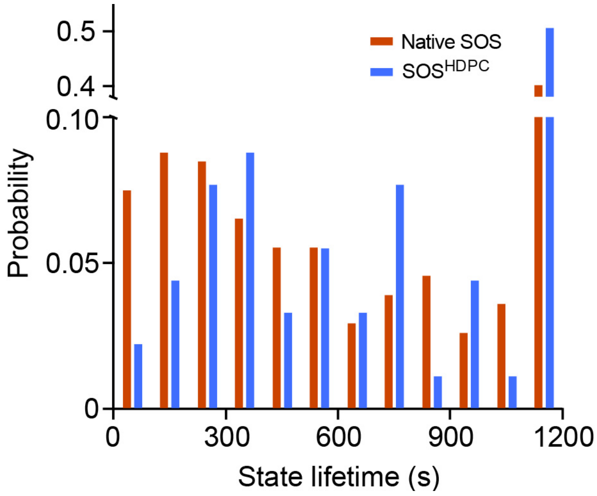

Supplementary Figure 8. Histogram of state lifetimes for native SOS and SOS^{HDPC}. State lifetimes were estimated from the duration of individual functional substates of SOS molecules. SOS activity was monitored for up to 1200 s. For detailed calculations of state lifetimes, see **Supplementary Fig. 5**.

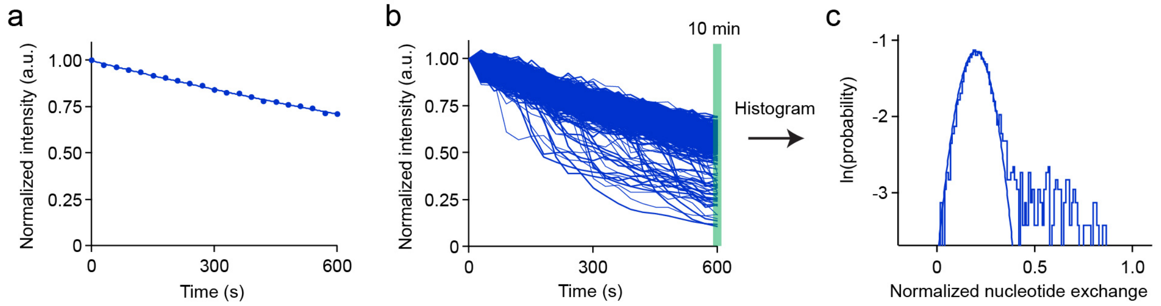

Supplementary Figure 9. Analysis approaches for steady state nucleotide exchange assays. As lysate is maintained in chambers throughout the reaction, all corrals experience multiple SOS molecules during the assays. Fluorescence decay rate of active corrals is a convolution of catalytic SOS turnover (k_{sos}), intrinsic Ras turnover (k_i) and photobleaching (k_p). Intrinsic Ras turnover and photobleaching kinetics was measured in the presence of 100 μM dark GTP in untransfected cell lysate as shown in (a). Then all traces were divided by the average intensity trace of untransfected lysate to remove intrinsic nucleotide exchange and photobleaching factors. The catalytic exchange kinetics of all corrals are shown in (b). Normalized nucleotide exchange distribution calculated from decrease in fluorescence intensity at 10 min is shown in (c).

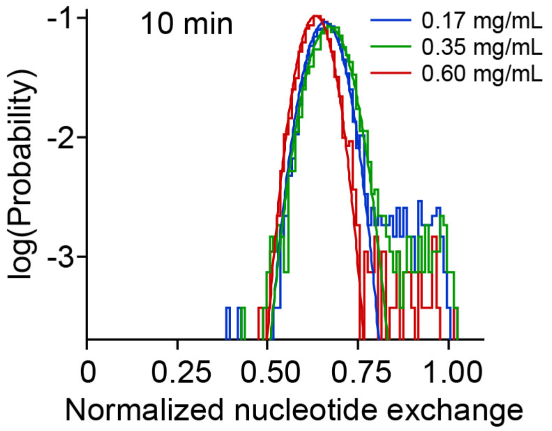

Supplementary Figure 10. Background lysate exhibits minimal effects on SOS activity. Steady state nucleotide exchange assays were performed with SOS^{Cat} supplemented with various concentrations of untransfected lysate up to ~0.6 mg/mL, which is the maximum lysate concentration used in the steady state nucleotide exchange assays. All solutions exhibit similar exchange activity (major populations fitted with Gaussian distribution). The probability of activated states (non-Gaussian populations) appears to show a slight dependency on lysate concentration. This could reflect some competition between SOS and other Ras binding proteins in the lysate. However, this dependency is much weaker compared to the effects of flanking domains shown in **Fig. 3** and **Supplementary Fig. 14**. Lipid composition (in mole percent): egg-PC/MCC-DOPE/DOPS = 94/3/3. SOS concentration: 2 nM. Ras surface density: 500 Ras/μm^2.

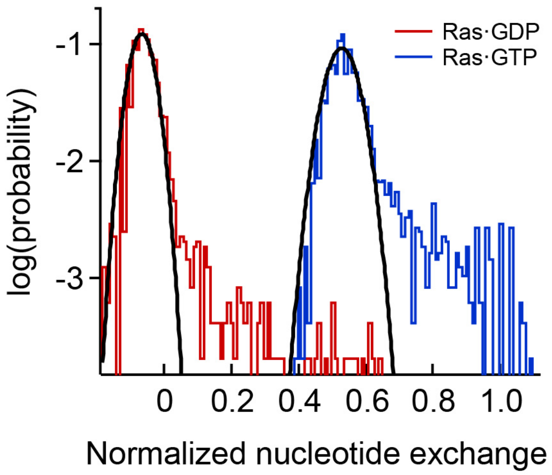

Supplementary Figure 11. Steady-state nucleotide exchange assay on Ras·GDP and Ras·GTP-functionalized membrane microarrays. When reacted with Ras·GDP, SOS^{HDPC} shows reduced nucleotide exchange ability for both random sampling and processive catalysis (the left-shift of Guassian population and the decreased occurrence of non-Gaussian population). Lipid composition (in mole percent): egg-PC/MCC-DOPE/DOPS = 94/3/3. SOS concentration: 1 nM. Ras surface density: 1500 Ras/μm^2.

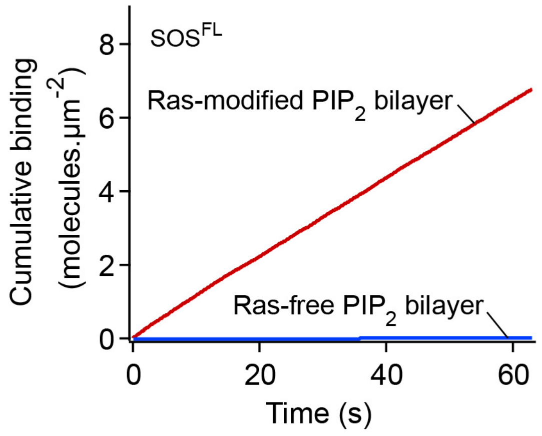

Supplementary Figure 12. Comparison of 1 nM SOS binding to Ras-modified or Ras-free bilayers. SOS^{FL} showed a significantly lower level of binding on a Ras-free bilayers. This demonstrates that the detectable binding events are attributed to interaction between Ras and SOS.

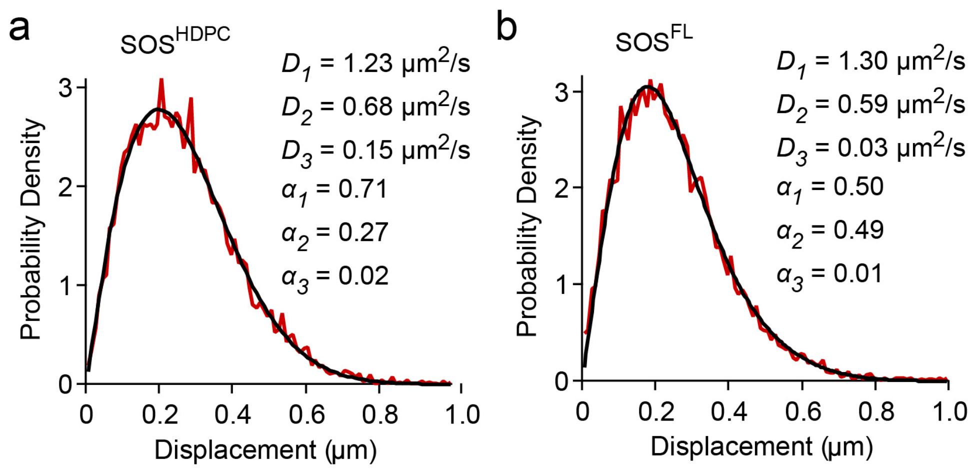

Supplementary Figure 13. Step size distribution analysis of SOS^{HDPC} and SOS^{FL} on Ras-modified PIP_2 bilayer reveals transient interactions between lipids and the N-terminal domains. SOS binds to one or two Ras molecules at either the catalytic or allosteric site, exhibiting two different diffusion coefficients (D_1 and D_2). In the presence of an additional interaction with PIP_2, three-species fitting of the step size distribution converges on two species with a negligible third population (shown as α_3 values). This implies that the timescale of the interactions between PIP_2 and N-terminal domains are shorter than the time resolution of the single-molecule tracking implemented here (~20 ms). Indeed, we did not observe detectable binding of SOS to the Ras-free PIP_2 bilayer in our single-molecule binding assays (**Supplementary Fig. 10**). However, PIP_2 markedly enhances SOS binding to Ras (**Fig. 4e**), suggesting that the transient interaction of PIP_2 is sufficient to induce a productive conformational rearrangement that increases the efficiency with which Ras·GTP accesses the SOS allosteric site. D_1 was fixed at the value obtained with two-species fitting (**Fig. 5**).

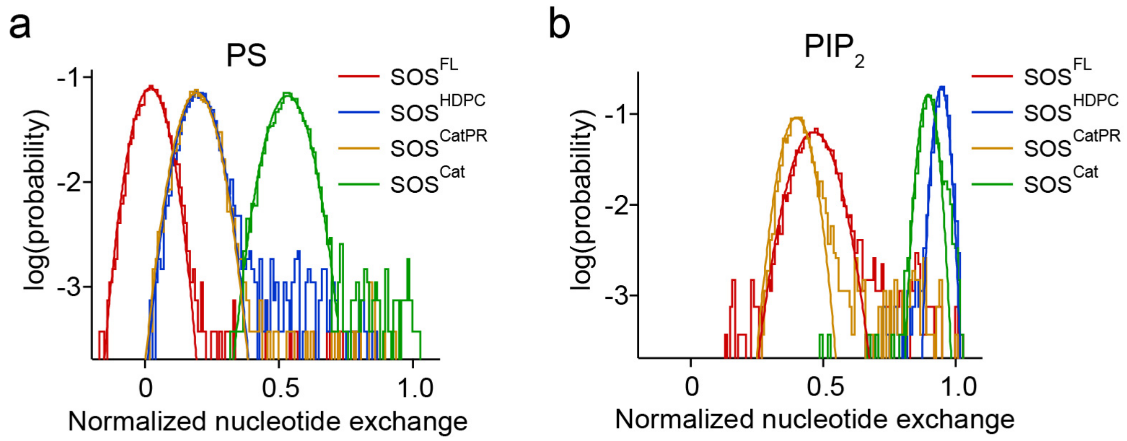

Supplementary Figure 14. PIP_2 strongly modulate activity of full-length SOS and SOS^{HDPC} by relieving autoinhibition of N-terminal domains. Steady state nucleotide exchange assays were performed in the presence of either (a) PS or (b) PIP_2. SOS^{HDPC} and SOS^{CatPR} show similar catalytic activity on the PS bilayer. However, PIP_2 selectively relieves autoinhibition of N-terminal domains in allosteric binding site, thus resulting in higher activity for SOS^{HDPC} with respect to SOS^{CatPR}. Full-length SOS also shows enhanced catalytic activity on the PIP_2 bilayer. However, full-length SOS is still partially autoinhibited by the PR domain and shows similar activity to SOS^{CatPR}. Lipid composition (in mole percent): (a) egg-PC/MCC-DOPE/DOPS = 94/3/3, (b) egg-PC/MCC-DOPE/PIP_2 = 94/3/3. SOS concentration: 2 nM. Ras surface density: (a) 500 Ras/μm^2, (b) 1200 Ras/μm^2. All samples have 0.64 mg/mL of background lysate.

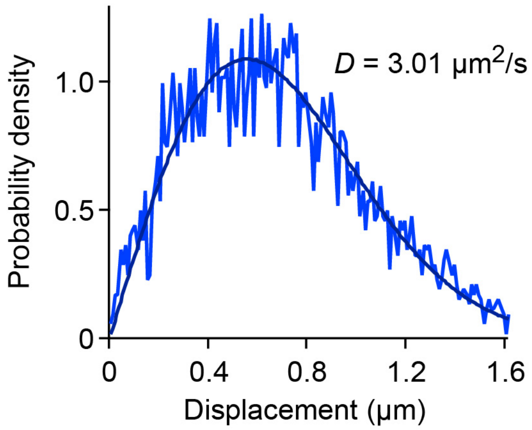

Supplementary Figure 15. Step size distribution of membrane-tethered Ras. A single-species model adequately describes Ras surface diffusion. Surface density of ~1800/um^2.

The retardation effects of the PR domain on the binding to Ras

**Ras**	$Ras^{(Y64A)}$	WT Ras	WT Ras
**Binding rate 1**<br>(PR domain-truncated SOS)	0.564<br>(SOS^{Cat})	0.526<br>(SOS^{Cat})	0.087<br>(SOS^{HDPC})
**Binding rate 2**<br>(PR domain-appended SOS)	0.113<br>(SOS^{CatPR})	0.078<br>(SOS^{CatPR})	0.012<br>(SOS^{FL})
**The effect of the PR domain**<br>(Binding rate 1/Binding rate 2)	**5.0**	**6.7**	**7.3**

Supplementary Table 1. The retardation effect of the PR domain was estimated by a ratio of the binding rates for two different SOS constructs, i.e. PR domain-truncated SOS divided by PR domain-appended SOS. Ras and SOS constructs used in each set of measurement are presented. Binding rates are in units of molecules$\cdot$nM$^{-1}\cdot\mu$m$^{-2}\cdot$ s^{-1}.

Supplementary References

1. Iversen, L. et al. Ras activation by SOS: Allosteric regulation by altered fluctuation dynamics. *Science* **345**, 50-54 (2014).
